# Walnut Lodge Hospital Hartford, Ct.

**Published:** 1894-12-29

**Authors:** 


### The Institutional Workshop.

### AN INSTITUTION FOR INEBRIATES.

### WALNUT LODGE HOSPITAL, HARTFORD, CT.

This institution was organised in 1878, for the express purpose of providing medical care and treatment for the better classes of alcohol and opium inebriates; also for those recent cases still in active life who for the first time realise the danger of their condition. The laws of the State give this hospital power to hold all voluntary cases for periods of either four months or one year. The latter must be by signature of the patient, and with his free will at the time. The first period of four months could be by simply requesting to be treated, either by himself, or the legal guardians, or those next-in-kin. If the patient ran away he could be returned by the police from any part of the State. If he thought he was unjustly confined he could appeal to the courts, who would order an examination, and decide if he was unjustly confined. Treatment: On admission a general examination was made, and the patient given a Turkish bath the first thing. In some cases spirits, wine, or beer are given at intervals, but in most of the cases all spirits are stopped at once. Various forms of chinchona, strychnine, carbonate of ammonia, acid phosphates, and occasionally bromide of sodium are given. Hot milk, lemonade, coffee, and carbonate waters are given freely for the first week. For extreme restlessness, baths, Turkish and hot showers, with salt rubs and exercises such as walks or work in the gymnasium. Calomel and Rochelle salts are alternated every two days for the first week. The arrangements of the hospital require every case to be prompt at meals three times a day; also at family prayers in the morning, and to retire at a regular hour at night. The diet is rich and wholesome, containing abundance of milk, fruit, and vegetables. Efforts are made to have special diets for special cases. Every case is examined and treated according to its condition and causation. Electricity is used in some cases, water douches and various baths in others daily. Sometimes vigorous exercises are ordered with full diet. Certain patients are driven out every day, and efforts directed to rouse up the mind and change the current of its activity. Billiards and games are encouraged. A music hall with piano and organ is open to all who can play or enjoy music. All patients are under the constant care of trained nurses and attendants, who give each one the services of a companion as long as they are quiet and cheerful, but when they become irritable they act as keepers and return them to the house at once, where they are given baths and restricted for a time. The plan of treatment is the same as is given in all hospitals for neurotics, and has for its central object the restoration of the brain and nervous system, and by psychical treatment of the mind the building up of new views and new conceptions. All cases remain from four to twelve months, and go away restored. About one hundred and twenty cases are received yearly, and at least one-third or more are cured or remain well after periods of seven or eight years, or as long as our statistics extend. Some cases who are true dipsomaniacs and periodics return for treatment often, either after a relapse or when on the verge of relapse. Such cases are received for different intervals of time, and go away restored. Some cases who are in active business or professional life come here after periods of prolonged overwork and exhaustion to avoid the risk of relapsing and using spirits again. A certain number of cases find by experience they can live here without the use of spirits, but will relapse elsewhere. This hospital is a private corporation, in which the superintendent, Dr. T. D. Crothers, is a large owner. An advisory board of medical men visit the hospital monthly and quarterly and decide all questions that are referred to them. The superintendent and assistant physician, with steward, a staff of attendants, and matron comprise the governing staff. All patients pay for medical care and board, from £3 to £6 a week. The *Quarterly Journal of Inebriety*, the oldest scientific publication on the question of inebriety, has been issued from this hospital under the care of Dr. Crothers from 1877. It is the organ of the American Association for the Study and Cure of Inebriety. This hospital is the oldest and largest private corporate asylum in America and much of its work has been published in the journal. It claims to have no specific plan of treatment, and regards the inebriate as a neurotic requiring restraint and building up of all the organism. Both the hospital and its superintendent, Dr. Crothers, enjoy the highest respect and esteem of the medical profession in America.

